# Adjusting for dependent comorbidity in the calculation of healthy life expectancy

**DOI:** 10.1186/1478-7954-4-4

**Published:** 2006-04-18

**Authors:** Colin D Mathers, Kim M Iburg, Stephen Begg

**Affiliations:** 1 Evidence and Information for Policy, World Health Organization, Geneva, Switzerland; 2 Division of Information, Evidence and Communication, World Health Organization, Regional Office for Europe Copenhagen, Denmark; 3 School of Population Health, University of Queensland, Brisbane, Australia

## Abstract

**Background:**

Healthy life expectancy – sometimes called health-adjusted life expectancy (HALE) – is a form of health expectancy indicator that extends measures of life expectancy to account for the distribution of health states in the population. The World Health Organization has estimated healthy life expectancy for 192 WHO Member States using information from health interview surveys and from the Global Burden of Disease Study. The latter estimates loss of health by cause, age and sex for populations. Summation of prevalent years lived with disability (PYLD) across all causes would result in overestimation of the severity of the population average health state because of comorbidity between conditions. Earlier HALE calculations made adjustments for independent comorbidity in adding PYLD across causes. This paper presents a method for adjusting for dependent comorbidity using available empirical data.

**Methods:**

Data from five large national health surveys were analysed by age and sex to estimate "dependent comorbidity" factors for pairs of conditions. These factors were defined as the ratio of the prevalence of people with both conditions to the product of the two total prevalences for each of the conditions. The resulting dependent comorbidity factors were used for all Member States to adjust for dependent comorbidity in summation of PYLD across all causes and in the calculation of HALE. A sensitivity analysis was also carried out for order effects in the proposed calculation method.

**Results:**

There was surprising consistency in the dependent comorbidity factors across the five surveys. The improved estimation of dependent comorbidity resulted in reductions in total PYLD per capita ranging from a few per cent in younger adult ages to around 8% in the oldest age group (80 years and over) in developed countries and up to 15% in the oldest age group in the least developed countries. The effect of the dependent comorbidity adjustment on estimated healthy life expectancies is small for some regions (high income countries, Eastern Europe, Western Pacific) and ranges from an increase of 0.5 to 1.5 years for countries in Latin America, South East Asia and Sub-Saharan Africa.

**Conclusion:**

The available evidence suggests that dependent comorbidity is important, and that adjustment for it makes a significant difference to resulting HALE estimates for some regions of the world. Given the data limitations, we recommend a normative adjustment based on the available evidence, and applied consistently across all countries.

## Introduction

Healthy life expectancy or health-adjusted life expectancy (HALE) is a form of health expectancy indicator which summarizes total life expectancy in terms of equivalent years of full health by taking into account the prevalence and severity distributions of health states in the population [1]. In the *World Health Report 2000*, the World Health Organization (WHO) reported for the first time on the average levels of population health for its 191 member countries using HALE [2,3].

Healthy life expectancy has previously been calculated for Australia, Canada and the United States using population survey data on disability [4-8]. Burden of disease analyses have also been used to calculate healthy life expectancy at global, regional and national levels [9,10]. In the burden of disease approach, the incidence, prevalence, duration and severity of disabling sequelae of diseases and injuries are estimated cause by cause for the population, for a comprehensive set of causes. The WHO estimates of HALE for Member States have been based on methods that combine available information from health interview surveys and from the Global Burden of Disease 2000 (GBD 2000) study [11,12].

Use of the Global Burden of Disease estimates of health state prevalences requires that these be added up across disease and injury causes. However, many people have more than one disease or injury, particularly at older ages. This comorbidity must be taken into account in adding up disease specific estimates if we are not to overestimate the average loss of health in the population. Additionally, the severity of health states associated with pairs of conditions, as measured by disability weights in the GBD 2000, may not simply be the sum of the two disability weights for the conditions. Its likely in many cases to be less than the sum, but in some cases there may be exacerbating effects on health states of having both diseases.

When HALE estimates were first published in the World Health Report 2000, adjustments were made for independent comorbidity as described below. The methods used were peer-reviewed during 2001 and 2002 by a Scientific Peer Review Group [13] which made a number of technical recommendations addressed in subsequent HALE calculations. In particular, methods were developed to take into account residents in health institutions and dependent comorbidity.

This paper describes the approach for dealing with dependent comorbidity. Dependent comorbidity refers to the situation where the probability of having a pair of diseases is greater than the product of the probabilities for each disease, reflecting common causal pathways (for example common risk factors causing both diabetes and heart disease) and also that one disease may increase the risk of another. The paper first presents a theoretical approach to adjusting for dependent comorbidity, then an operationalization of this approach using analysis of available empirical data, and finally presents sensitivity analyses for certain assumptions required by the method.

### Previous approaches for dealing with comorbidity

Barendregt and Bonneux have carried out a sensitivity analysis of health adjusted life expectancy to comorbidity between five diseases (ischaemic heart disease, congestive heart failure, cerebrovascular disease, lung cancer and chronic obstructive pulmonary disease). They assumed independent comorbidity: the probability of having two diseases is the product of the probability or prevalence of each [14]. Through a sensitivity analysis, they concluded that the overall effect of comorbidity on estimated healthy life expectancy is small, and that simple assumptions on comorbidity disability weights will be acceptable, because the impact on HALE estimates will be minor.

**Figure 1:**
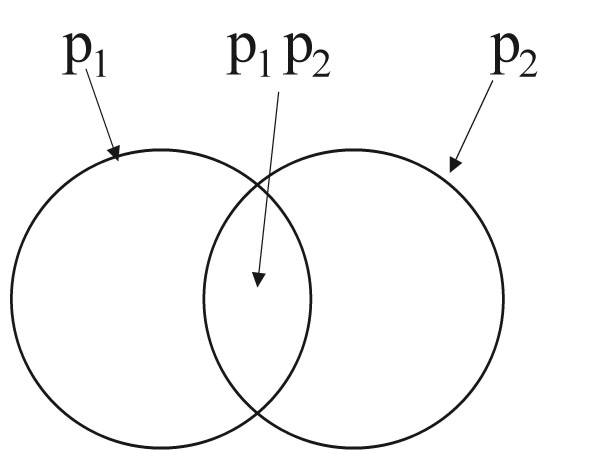
Independent comorbidity.

For the first HALE calculations reported in the World Health Report 2000, all comorbidity between disease and injury causes was also assumed to be independent comorbidity [3]. Independent comorbidity is the situation where the probability of having two (comorbid) conditions is assumed to equal the product of the probabilities for having each of the diseases:

*P*_1+2 _= *P*_1_+*P*_2 _= 1(1-*P*_1_)× (1-*P*_2_)     (1)

where *p*_*1*+*2 *_is the prevalence of the two comorbid diseases 1 and 2, *p*_*1 *_is the prevalence of disease 1 and *p*_*2 *_the prevalence of disease 2. Independent comorbidity is illustrated in Figure 1.

The proportion of years lived at each age in equivalent good health, required for the calculation of HALE (see Methods section) is estimated in the burden of disease approach using the prevalence YLD per capita for each cause:

*PYLD*_*i *_= *DW*_*i*_× *p*_*i *_    (2)

where *PYLD*_*i *_is the prevalence YLD for cause *i*, *DW*_*i *_is the disability weight for cause *i*, and *p*_*i *_is the prevalence rate per capita for cause *i*. Ignoring comorbidity for the moment, the total PYLD per capita summed across all causes represents the average lost years of equivalent full health per capita (at a given age) and one minus this quantity represents the proportion of years lived at that age in equivalent good health.

The simplest approach to estimating the disability weight for the combined conditions 1 and 2 is to assume that the health state valuations (1-disability weight) are multiplicative, so that the combined weight is more severe than the weight for either condition on its own, and remains bounded by 0 and 1 [10]. If the disability weight for the combined conditions 1 and 2 is given by:

*DW*_*1*+*2 *_= *1*-(*1*-*DW*_*1*_) × (*1*-*DW*_*2*_)     (3)

then the two calculations given by equations (1) and (3) can be combined into a single calculation for the combined prevalence YLD as follows:

*PDLD*_*1*+*2 *_= *1*-(*1*-*PYLD*_*1*_) × (*1*-*PYLD*_*2*_)     (4)

This formula can be generalized to deal with more than two causes as follows:



where Π denotes the product operator.

### Adjusting for dependent comorbidity

For the second round of HALE estimates published in the World Health Reports 2001 [15], dependent comorbidity was explicitly taken into account for Vitamin A deficiency and iron-deficiency anaemia (50% and 25% respectively assumed to be comorbid with protein-energy malnutrition), for diabetes with cardiovascular disease, and for chronic obstructive pulmonary disease with cardiovascular disease (comorbidity estimated from smoking prevalence data as common cause) [12].

**Figure 2:**
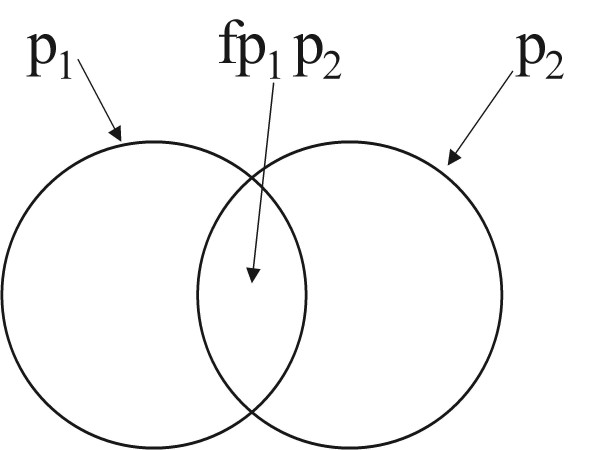
Dependent comorbidity.



Following the scientific peer review [13], we developed a more general and comprehensive approach to dealing with dependent comorbidity, as described in this paper. The approach outlined above for adjusting the sum of PYLD for independent comorbidity can be generalized to allow for dependent comorbidity. Let us define the comorbidity factor *f *for two conditions as follows (see Figure 2):

Thus an *f *factor of 2 would indicate that the prevalence of conditions 1 and 2 together is twice as common as would be expected if the occurrence of the two conditions was independent. An *f *factor of 1 would indicate that the comorbidity is independent. Using this *f *factor, and the same assumption as above about the disability weight for the combined conditions, we can calculate the PYLD for conditions 1 and 2 as follows:

*PYLD*_1+2 _= *PYLD*_1_+*PYLD*_2 _- *f*_1+2 _× *PYLD*_1 _× *PYLD*_2_

= *PYLD*_1 _+ (1-*f*_1+2 _× *PYLD*_1_) × *PYLD*_2 _    (7)

where *f*_*1*+*2 *_denotes the *f *factor for the two conditions 1 and 2.

When there are more than 2 causes, calculation of the total PYLD for all causes using the above approach would involve all pairwise *f *factors plus potential terms for higher order comorbidity between 3 or more conditions. This complexity can be avoided by taking a sequential approach to the calculation of the total PYLD, where at each step the PYLD for condition j+1 is added to the total PYLD for conditions 1 to j, and the required *f *factor is that for condition j with the total prevalence for conditions 1 to j:

*PYLD*_1+...+(*j*+1) _= *PYLD*_1+...+*j *_+ (1-*f*_(1+...+*j*)+(*j*+1) _× *PYLD*_1+...+*j*_) × *PYLD*_*j*+1 _    (8)

## Methods

### Analysis of dependent comorbidity reported in national health surveys

Data from five large national health surveys in Australia, United States of America, Denmark and Belgium (see Table 1) were analysed by age and sex to estimate "dependent comorbidity" *f *factors for pairs of conditions. These conditions were self-reported by survey respondents. To enable results for *f *factors to be compared and pooled across surveys, it was necessary to group self-reported conditions into broad disease and injury categories to avoid problems arising from differences in finer disease labels used. It was also decided that too many disease categories would be inappropriate given sample sizes and the low prevalences of many specific conditions. The final set of categories used were cardiovascular conditions and diabetes, chronic respiratory conditions, musculoskeletal conditions, nervous system conditions, mental disorders, and other conditions (including infectious diseases and injuries and their sequelae).

**Table 1:** Five population health surveys used in analysis. Year of survey, sample size, and abbreviation used in other tables.

Abbreviation	Survey	Year	Sample size
**AUSNHS**	Australian National Health Survey	1995	53,700
			
**AUS NMHS**	Australian National Survey of Mental Health and Wellbeing	1997	10,600
			
**US NHIS**	US National Health Interview Survey	2000	32,375
			
**Denmark**	Danish Health and Morbidity Survey	1994	6,786
			
**Belgium**	Belgian Health Interview Survey	1997	7,967

The Australian National Health Survey 1995 [16] was conducted on a multistage, cluster sample of households in all states and territories of Australia. Information was obtained by personal interviews of 53,751 persons. The survey contains detailed information on health status, including self-reports of recent and long-term medical conditions experienced by respondents. The Australian National Survey of Mental Health and Wellbeing 1997 [17,18] provided information from personal interviews of 10,600 persons aged 18 years or more on the prevalence of selected major mental disorders, and on chronic physical conditions and disability. The response rate was 78%.

The US National Health Interview Survey 2000 [19] collected self-reported information on health status and illness conditions. We utilized information from respondents 18 years and older. The response rate was 82.6% and the adult sample size was 32,374 persons.

The Danish Health and Morbidity Survey 1994 [20,21] contains information from 4,668 persons obtained from a representative national sample plus 2,119 persons from two Danish counties collected in the same year, resulting in a total sample of 6,787 adult persons over 16 years of age. The overall response rate to the interviews was 79%. Data were collected through a 45 minute interview together with a self-administered questionnaire to be mailed back within two weeks.

The Belgian Health Interview Survey 1997 [22] consisted of three parts: 1) a household survey for household and demographic information, 2) a self-administrated questionnaire including questions on health complaints and symptoms, and mental health, and 3) a face-to-face interview including questions on chronic diseases, limitations and handicaps. The survey was of 7,967 persons 15 years and older in Belgium's three regions the Flemish Region, the Walloon Region and the Brussels Region. The overall response rate was 60.5%.

### Adjustment of HALE for dependent comorbidity

HALE estimates for WHO Member States have been carried out using Sullivan's method [23], which requires three inputs: life tables and prevalences of various states of health together with appropriate severity weights. The development of WHO life tables and of health state severity weights is described elsewhere [11,24,25], we focus here on the estimation of health state prevalences.

The health state valuations used in HALE calculations represent average population assessments of the overall health levels associated with different states. They range from 1 representing a state of good or ideal health to 0 representing states equivalent to being dead. Sullivan's method requires estimates of age- and sex-specific average health state valuations for the population for the specified time period (usually a calendar year). We use the notation *H*_*x*,*s *_to denote the population average health state valuation for sex *s *and age group *x*. If *L*_*x*,*s *_represents the total life table years lived by sex *s *in the age range corresponding to *x*, then *HALE*_*a*,*s *_at exact age *a *is calculated by summing the healthy years of life lived for each age group from a onwards and dividing by the number of life table survivors *l*_*a*,*s *_at exact age a:



In calculating HALE for the World Health Report 2000, WHO carried out an analysis of 62 representative population health surveys which revealed substantial problems with comparability of self-report health status and disability data [3]. As a result disability prevalence estimates from the GBD 2000 were used to adjust for biases in self-report data; the independent information on levels of population health provided by the health surveys was thus quite limited.

WHO undertook a Multi-Country Survey Study on Health and Responsiveness (MCSS) in 2000 and 2001 in collaboration with Member States using a standardized health status survey instrument together with new statistical methods for to adjust for biases in self-reported health [26-29]. These new data, together with comprehensive analyses of epidemiological data for all regions of the world from the GBD 2000, were used to calculate healthy life expectancy for WHO Member States for 2002 using methods explicitly developed to maximise comparability across countries. These methods are summarized below and described in more detail elsewhere [11,12].

Because the MCSS surveys were carried out in only 61 Member States, a three-stage strategy was used to obtain comparable health state prevalences for all 192 Member States. Firstly, data from the MCSS were used to make independent estimates of *H*_*x*,*s *_by age and sex for 58 countries (three were excluded due to survey quality issues). The MCSS survey samples did not include older people resident in nursing homes or other health institutions. Because these people will generally have worse health than those resident in households, adjustments were made to the *H*_*x*,*s *_estimates to account for the older population who were resident in health institutions [11].

Secondly, data from the GBD 2000 were used to estimate *H*_*x*,*s *_by age and sex for all 192 countries for the year 2002. The GBD estimated years lived with disability (YLD) for 135 major causes, for 17 sub-regions of the world [30]. The GBD analyses were used to prepare estimates of mortality and burden of disease for each WHO Member States for the year 2002. Mortality estimates were based on analysis of latest available national information on levels of mortality and cause distributions. YLD estimates were based on the GBD analyses of incidence, prevalence, duration and severity of conditions for the relevant epidemiological subregion, together with national and subnational level information available to WHO [31].

As well as the standard incidence-based YLD, prevalence-based YLD rates were calculated for each cause, as given by equation (2). For the original HALE estimates published in 2000 and 2001, the prevalence YLD were added across causes with adjustment for independent comorbidity as given by equation (5). For the later estimates published in World Health Reports in 2003 and 2004 [32,33], adjustments for dependent comorbidity were carried out using *f *factors from analysis of the five surveys described above.

The *f *factors from the survey analyses were compared and averaged to give a final set of dependent comorbidity factors used for adjusting the summation of PYLD across causes for each country. The adjustments were carried out using the cumulative method outlined above and the following sequence of cause groups: cardiovascular disease and diabetes, chronic respiratory diseases, musculoskeletal diseases, sight or hearing loss, Group 1 conditions (communicable, maternal, perinatal and nutritional conditions), injuries, other diseases, neurological diseases, mental disorders.

For all WHO HALE calculations irrespective of method of comorbidity adjustment, the final prevalence YLD rate per capita summed across all causes was used to estimate average "prior" health state valuations for the populations of WHO Member States:



Because there is potential measurement error in severity-weighted health state prevalences derived from both household surveys and epidemiological estimates, posterior estimates of prevalence for the survey countries were calculated as weighted averages of the GBD-based prevalences and the survey prevalences:



where the weights *w*_*x*,*s *_were based on the estimated relative uncertainties of the GBD and survey-based population average health state valuations *H*_*x*,*s *_for sex *s *and age group *x*. The relationship between the GBD-based  and the posterior  was estimated for the survey countries using ordinary least squares regression and the results used to adjust the  for the non-survey countries. This ensured that the use of the survey data did not introduce a differential between survey and non-survey countries, and allowed the survey evidence to be indirectly taken into account in making the best possible estimates for non-survey countries.

### Sensitivity analyses

A sensitivity analysis was carried out of the impact of the magnitude of the *f *factor on the adjustment to total PYLD and hence to estimates of *H*_*x*,*s*. _For this analysis, the f factors were assumed to be the same across all sequential condition groups and varied from 1 (independent comorbidity) through to 5.

As noted above, self-reported conditions were grouped into broad disease and injury categories for the analysis of *f *factors from the survey data. A second sensitivity analysis was carried out to examine the sensitivity of the HALE estimates to the sequencing of the disease and injury groups for the dependent comorbidity adjustments.

## Results

Table 2 shows the *f *factors calculated from the five survey datasets. Differences in the comprehensiveness in self-reported conditions collected in the various surveys meant that *f *factors for some categories could not be calculated for some of the surveys. However they have been included in the table for comparison. There was surprising consistency in the *f *factors across the five surveys, both in terms of the magnitudes and the age patterns. The *f *factors were typically around 1.5 to 2 at older ages, around 3 to 5 at middle ages and higher at younger ages (where prevalences are typically low). An *f *factor of 5 at middle ages signifies that the prevalence of the comorbid pair of conditions is five times higher than would be expected by chance alone based on the observed prevalences for each of the conditions considered separately,

**Table 2:** Comorbidity factors from five population surveys. Comorbidity factors for cumulative cause groups by age and sex. The comorbidity factor *f *is computed using equation (6) as the prevalence of persons reporting diseases in the two cause groups (*p*_*1*+*2*_) divided by the two prevalences *p*_*1 *_and *p*_*2 *_for each cause group considered independently: *f *= *p*_*1*+*2*_/(*p*_*1 *_× *p*_*2*_)

		Males					Females				
			
Survey	Combinations	< 30	30–44	45–59	60 –74	75+	< 30	30–44	45–59	60–74	75+
**AUSNHS**	Cardiovascular + respiratory	114.77	43.84	7.93	4.05	7.36	48.36	16.14	9.72	6.03	6.80
	Previous + musculoskeletal	5.91	8.73	5.12	2.84	4.27	5.77	5.68	4.89	3.84	4.31
	Previous + other	3.87	2.58	1.72	1.40	1.72	3.87	2.64	1.78	1.38	1.65
	Previous + nervous	4.88	3.47	2.25	1.64	2.13	4.72	3.28	1.94	1.47	1.92
	Previous + mental	2.93	2.01	1.43	1.20	1.45	2.87	1.98	1.47	1.25	1.43
											
**AUS NMHS**	Cardiovascular + respiratory	--	--	9.66	3.76	4.05	41.99	35.78	14.62	6.12	4.53
	Previous + musculoskeletal	37.19	33.12	8.47	3.47	3.52	27.62	21.66	11.54	5.13	4.21
	Previous + other	23.44	15.85	4.03	2.07	2.21	19.34	10.31	3.56	1.91	1.89
	Previous + mental	4.76	4.83	2.29	1.44	1.73	4.25	3.93	2.09	1.37	1.46
											
**US NHIS**	Cardiovascular + respiratory	9.97	4.10	1.80	2.59	1.77	8.14	4.12	2.20	1.42	2.37
	Previous + musculoskeletal	7.07	3.55	1.64	2.38	1.56	4.78	3.07	1.89	1.31	2.24
	Previous + other	2.00	2.00	1.56	1.22	1.22	1.92	1.92	1.57	1.27	1.27
	Previous + mental	1.84	1.84	1.46	1.15	1.15	1.65	1.65	1.39	1.17	1.17
											
**Denmark**	Cardiovascular + respiratory	32.71	32.71	14.05	6.04	6.04	41.56	41.56	15.63	5.87	5.87
	Previous + musculoskeletal	13.49	13.49	5.76	2.46	2.46	14.38	14.38	7.43	3.84	3.84
	Previous + other	4.15	4.15	2.61	1.64	1.64	4.35	4.35	2.66	1.63	1.63
	Previous + nervous	4.08	4.08	2.63	1.69	1.69	4.46	4.46	2.73	1.67	1.67
	Previous + mental	3.12	3.12	2.09	1.40	1.40	3.20	3.20	2.05	1.32	1.32
											
**Belgium**	Cardiovascular + respiratory	33.02	33.02	11.66	4.12	4.12	43.21	43.21	15.74	5.73	5.73
	Previous + musculoskeletal	11.06	11.06	5.28	2.52	2.52	11.12	11.12	6.31	3.58	3.58
	Previous + mental	5.90	5.90	3.27	1.81	1.81	5.72	5.72	3.48	2.12	2.12

A final set of *f *factors were calculated by averaging the *f *factors across surveys and applying these *f *factors to a slightly more detailed set of sequential cause categories. The dependent comorbidity factors shown in Table 3 were used for all Member States to adjust for dependent comorbidity in summation of prevalence YLD across all causes, as there was insufficient evidence to justify use of different *f *factors in different regions of the world.

**Table 3:** Final dependent comorbidity factors. Dependent comorbidity factors *f *used in the calculation of all-cause PYLD per capita. The comorbidity factor *f *is defined as the prevalence of persons with comorbid diseases in the two cause groups divided by the two prevalences for each cause group considered independently, *f *= *p*_*1*+*2*_/(*p*_*1 *_× *p*_*2*_).

	**Males**								**Females**							
		
**Condition pair**	**0–4**	**5–14**	**15–29**	**30–44**	**45–59**	**60–69**	**70–79**	**80+**	**0–4**	**5–14**	**15–29**	**30–44**	**45–59**	**60–69**	**70–79**	**80+**
CVD + diabetes	4.0	4.0	4.0	4.0	4.0	4.0	4.0	4.0	4.0	4.0	4.0	4.0	4.0	4.0	4.0	4.0
+ respiratory*	26.2	26.2	26.2	15.1	5.9	3.7	3.7	3.6	25.0	25.0	25.0	16.1	7.4	3.7	4.0	4.2
+ Musculoskeletal	10.9	10.9	10.9	9.0	3.9	2.7	2.6	2.5	9.1	9.1	9.1	7.6	4.6	2.8	3.0	3.3
+ Sight or hearing loss	5.2	5.2	5.2	4.3	2.3	1.6	1.6	1.7	5.0	5.0	5.0	3.9	2.3	1.5	1.6	1.6
+ Group1***	5.2	5.2	5.2	4.3	2.3	1.6	1.6	1.7	5.0	5.0	5.0	3.9	2.3	1.5	1.6	1.6
+ Injuries	4.9	4.9	4.9	4.2	2.6	1.7	1.8	1.9	4.8	4.8	4.8	4.0	2.4	1.6	1.7	1.9
+ Other diseases	5.2	5.2	5.2	4.3	2.3	1.6	1.6	1.7	5.0	5.0	5.0	3.9	2.3	1.5	1.6	1.6
+ Neurological	4.5	4.5	4.5	3.8	2.3	1.6	1.5	1.5	4.5	4.5	4.5	3.8	2.3	1.6	1.5	1.5
+ Mental disorders	4.1	4.1	4.1	3.9	2.2	1.4	1.5	1.6	3.9	3.9	3.9	3.5	2.2	1.5	1.5	1.5

*The first condition of each pair is the cumulative prevalence of having one or more of the conditions in preceding rows.

*** Communicable diseases, maternal and perinatal conditions and nutritional deficiencies.

Figure 4 shows the results for males aged 80 years and over for a typical developing country. Simple addition of PYLD across causes without any adjustment for comorbidity results in a total PYLD of 0.85 (an average health state equivalent to severe Alzheimer's disease or quadriplegia). Adjustment for independent comorbidity (*f *= 1) reduces this to around 0.59, still a health state more severe than blindness. As the *f *factor increases up to 5, the average health state valuation reduces to around 0.33, not as severe but still a state of considerable health problems.

**Figure 3:**
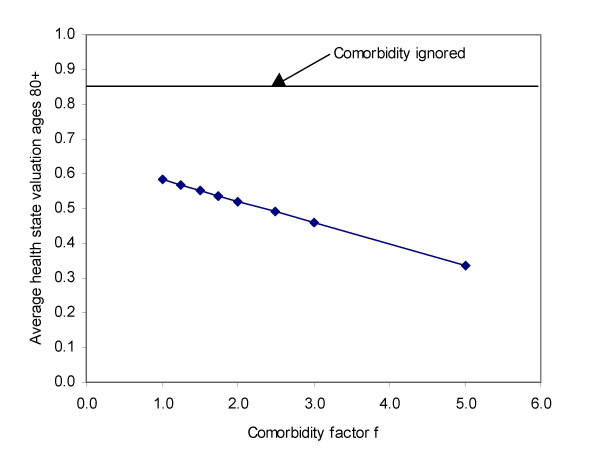
**Sensitivity of average health state valuation to dependent comorbidity factor f**. Variation of average health state valuation for age group 80 and over with assumed value of dependent comorbidity factor *f *(same factor assumed for all disease pairs): example for males in a developing country.

**Figure 4:**
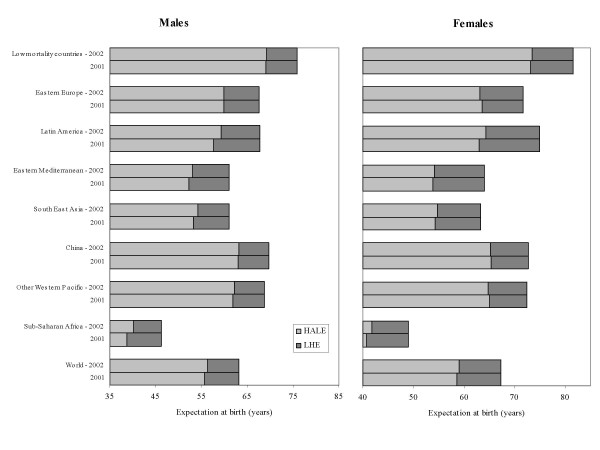
**A comparison of regional healthy life expectancy at birth in 2002 calculated with and without dependent comorbidity adjustment**. Healthy life expectancy (HALE) and Lost Health Expectancy (LHE) at birth in 2002, calculated using as inputs the GBD estimated PYLD for 2002 with dependent comorbidity adjustments (bars labelled 2002) and the GBD estimated PYLD for 2001 with independent comorbidity adjustments (bars labelled 2001).

The overall effect of the introduction of the dependent comorbidity adjustment is a reduction across all countries in the total PYLD rate per capita by age and sex from the GBD 2000 country estimates, and hence an increase in healthy life expectancy. The amount of change varies somewhat across regions. The improved estimation of dependent comorbidity resulted in reductions in total PYLD per capita ranging from a few per cent in younger adult ages to around 8% in the oldest age group (80 years and over) in developed countries and up to 15% in the oldest age group in the least developed countries.

Figure 4 illustrates the impact of the dependent comorbidity adjustment on regional healthy life expectancy at birth in 2002. The bars labelled 2002 were calculated using the 2002 country life tables, the health state valuations from the MCSS surveys and the Global Burden of Disease estimated PYLD for 2002 with dependent comorbidity adjustments. The bars labelled 2001 show the same healthy life expectancies calculated with the Global Burden of Disease inputs replaced by estimated PYLD for 2001 with independent comorbidity adjustments. The differences are slight for low mortality countries, Eastern Europe, China and the Western Pacific. In contrast, the dependent comorbidity adjustments increase healthy life expectancy by around 0.5 to 1.5 years for Latin America, South East Asia and Sub-Saharan Africa.

The analysis of the surveys was repeated to calculate *f *factors for sequentially cumulative cause groups using three different orderings of the cause groups, in order to test the sensitivity of the results to the assumed order. The three orders are shown in Table 4.

**Table 4:** Three orderings of cumulative condition groups for sensitivity analysis. Three orderings of cumulative condition groups for analysis of dependent comorbidity factors and sensitivity of dependent comorbidity adjustments to condition ordering.

Order 1	Order 2	Order 3
Cardiovascular + respiratory	Musculoskeletal + nervous system	Mental + respiratory
Previous + musculoskeletal	Previous + Cardiovascular	Previous + musculoskeletal
Previous + injuries	Previous + respiratory	Previous + Cardiovascular
Prev + nervous system	Previous + Group 1	Prev + nervous system
Previous + Group 1	Previous + other	Previous + Group 1
Previous + other	Previous + mental	Previous + other
Previous + mental	Previous + injuries	Previous + injuries

The age standardized PYLD rate per capita (a number between 0 and 1 corresponding to the average health state valuation *H*) is shown in Table 5 for the three orderings for a developing country (Ghana) and a developed country (Sweden). Dependent comorbidity adjustment of any kind makes a big difference to the total PYLD rate, there are also some smaller differences between the results for the three orderings. The corresponding differences in HALE at birth are shown in the 2 right hand columns of Table 5. Adjustment for dependent comorbidity increases HALE by around 1 year for both males and females in Ghana and for females in Sweden, and by around 0.5 years for males in Sweden. The ordering of the condition groups in carrying out the adjustment makes some difference also, with a range of around 0.3 years for males in Sweden and females in Ghana, and around 0.6 years for males in Ghana and females in Sweden.

**Table 5:** Sensitivity analysis of ordering of cumulative condition groups. Sensitivity of estimated age-standardized average health state valuation *H *and of estimated HALE at birth to three orderings of cumulative condition groups for dependent comorbidity adjustments for a developed country and a developing country.

	Age-standardized average health state valuation *H*		HALE at birth (years)	
		
	Male	Female	Male	Female
**Ghana**				
Independent comorbidity	0.151	0.096	46.8	48.9
Order 1	0.134	0.089	47.8	49.9
Order 2	0.128	0.088	48.1	50.0
Order 3	0.128	0.085	48.5	50.3
				
**Sweden**				
Independent comorbidity	0.066	0.044	70.5	72.6
Order 1	0.062	0.042	70.9	73.3
Order 2	0.060	0.041	71.0	73.6
Order 3	0.056	0.039	71.2	73.9

## Discussion and conclusion

Previous HALE calculations based on condition-specific data have made comorbidity adjustments on the assumption that the probability of occurrence of different diseases in one individual are statistically independent. This paper has presented a general method for making comorbidity adjustments taking into account dependent comorbidity, that is, the situation where pairs of disease occur with greater frequency than would be they case if they were independent. Quantification of dependent comorbidity was based on an analysis of self-reported data from five large national health surveys.

The available evidence suggests that dependent comorbidity is important, and that adjustment for it makes a significant difference to resulting HALE estimates for some regions of the world. The improved estimation of dependent comorbidity resulted in reductions in total PYLD per capita ranging from a few per cent in younger adult ages to around 8% in the oldest age group (80 years and over) in developed countries and up to 15% in the oldest age group in the least developed countries. This has resulted in an upward adjustment in the HALE estimates for WHO Member States reflecting the consistent evidence from health surveys that dependent comorbidity is common for most conditions.

To date, this evidence is based on health surveys from developed countries, and it will be important to extend this analysis to health surveys in developing countries. However, in extending the analysis, it will be difficult to take into account the known differences in reporting behaviour for illnesses and impairments between people in developing and developed countries [34,35]. Many surveys have shown that people in developing countries report much lower prevalences of illnesses and impairments. In part this is due to lower access to health services resulting in less awareness of illnesses, and in part to difference implicit standards for labelling and reporting health problems. Such differences will make it difficult to interpret whether differences in *f *factors between self-report data in developing and developed countries are real or are a result of differences in reporting behaviours.

We have chosen to apply the *f *factors, derived in our analysis of five large surveys in four countries, to all countries as a normative evidence-based adjustment for dependent comorbidity as it seems unlikely that unbiased evidence on differences in dependent comorbidity across countries and regions is feasible in the near future.

The sensitivity to order of adjustment, noted above in the sensitivity analysis, is also a result of using self-report data from surveys for the estimation of *f *factors. If a consistent set of disease prevalences were used for the estimation of *f *factors and for the calculation of PYLD then the sequential cumulative adjustment method must be independent of order (this can be shown mathematically). Because we are using *f *factors derived from self-report survey data, and applying them to GBD estimates of prevalences derived from synthesis of epidemiological data from population studies using carefully defined case definitions for diseases and their sequelae, the results may depend on the order of adjustment. This is because the GBD prevalences are not necessarily consistent with the survey self-report prevalences.

The only way to properly solve this problem is to carry out a very large population survey in which prevalences are ascertained using appropriate diagnostic tests and GBD case definitions. This would be so expensive as to almost certainly never be likely to be carried out. It would certainly be possible to obtain more rigorous data on dependent comorbidity for some selected condition pairs, for example from countries with comprehensive person-based medical records, but this would not help us solve the full comorbidity adjustment problem.

The order that we have chosen for the adjustment of HALE gives an increase in HALE at the lower end of the range. In other words, it is a more conservative adjustment than given by the other orderings. If it is possible to obtain analyses of *f *factors for condition pairs based on more objective case definitions consistent with those used in the GBD 2000, then it might be possible to take these into account in adjusting for dependent comorbidity in HALE. The analyses reported here could be used to make an initial determination of the most important condition pairs for dependent comorbidity adjustment (this would take into account prevalence, severity and best estimate of *f *factors). Such a short list of important pairs could then be used to search for empirical evidence to improve the adjustments for these pairs.

Another area requiring further investigation is the estimation of disability weights for comorbid pairs of conditions. The usual techniques for eliciting health state valuations either present valuers with a pure health state description (using the Euroqol or HUI or similar multi-domain health state description tool) or with a disease label. Sometimes the disease label is supplemented with a health state description [36] or the respondent is asked to write the health state description for the disease label they are valuing (MCSS). Extending these approaches to comorbid pairs of conditions seems to present a lot of difficulty. The respondents are either guessing what the impact of the pair of conditions is on the health state profile, or there is a need for that to be provided from empirical studies.

A number of studies have examined the impact of comorbidity on overall levels of disability or functioning, usually for a selected small group of conditions. Although some of these also provide information on the probability of comorbidity for condition pairs, this has been a less obvious focus of research relating to comorbidity.

One large study of the impact of comorbidity of common impairments in older people on Activities of Daily Living (ADL) and Instrumental Activities of Daily Living (IADL) found that only a few combinations including vision and hearing loss acted to further exacerbate the effects of other impairments on disability [37]. A number of studies in Mexican-Americans, Americans, Canadians and Koreans have found that depression and comorbid medical conditions interact to increase the probability of depression and to reduce the health-related quality of life [38-44]. Certain physical conditions have also been found to be associated with a significantly increased likelihood of panic attacks [45,46].

A recent Dutch study of 1,673 non-institutionalized chronic disease patients found synergistic effects of combinations of diabetes, cardiovascular disease and chronic respiratory disease with a higher risk of physical disability than could be expected from their separate effects [47]. However, while these types of studies tell us that the disability associated with a comorbid state may be greater than the disability associated with either condition, they have not addressed the issue of whether the disability weights would be additive or sub-additive, as has been assumed in the methods outlined above. In the absence of such studies, the multiplicative assumption used here seems a reasonable step.

Barendregt and Bonneux concluded in their earlier paper that ignoring comorbidity is an attractive option because of the difficulty of bring empirical data to bear and the complex adjustments required, and that simple assumptions will probably serve because the impact on HALE estimates is minor [14]. We have shown that the available evidence suggests that dependent comorbidity is important, and that adjustment for it makes a significant difference to resulting HALE estimates. Given the data limitations, a normative adjustment based on the available evidence, but applied consistently across all countries, seems to be the most justifiable approach. This is the approach that has been taken for the calculation of HALE for WHO Member States in recent World Health Reports [32,33].

## Competing interests

The author(s) declare that they have no competing interests.

## Authors' contributions

CM conceived the approach to adjustment for dependent comorbidity, KMI and SB carried out analyses of the five health surveys, CM and KMI undertook sensitivity analyses, calculations of healthy life expectancies and initial drafting of the paper. All three authors contributed to the writing of the paper.
